# Production of recombinant AAV vectors encoding insulin-like growth factor I is enhanced by interaction among AAV *rep *regulatory sequences

**DOI:** 10.1186/1743-422X-6-3

**Published:** 2009-01-07

**Authors:** Shuiliang Shi, Scott A Mercer, Robert Dilley, Stephen B Trippel

**Affiliations:** 1 Department of Orthopaedic Surgery, Indiana University School of Medicine, Indianapolis, IN, USA; 2 Department of Anatomy and Cell Biology, Indiana University School of Medicine, Indianapolis, IN, USA; 3 Indiana State Police Laboratory, 550 West 16^th ^Street, Indianapolis, IN 46202. USA

## Abstract

**Background:**

Adeno-associated virus (AAV) vectors are promising tools for gene therapy. Currently, their potential is limited by difficulties in producing high vector yields with which to generate transgene protein product. AAV vector production depends in part upon the replication (Rep) proteins required for viral replication. We tested the hypothesis that mutations in the start codon and upstream regulatory elements of Rep78/68 in AAV helper plasmids can regulate recombinant AAV (rAAV) vector production. We further tested whether the resulting rAAV vector preparation augments the production of the potentially therapeutic transgene, insulin-like growth factor I (IGF-I).

**Results:**

We constructed a series of AAV helper plasmids containing different Rep78/68 start codon in combination with different gene regulatory sequences. rAAV vectors carrying the human IGF-I gene were prepared with these vectors and the vector preparations used to transduce HT1080 target cells. We found that the substitution of ATG by ACG in the Rep78/68 start codon in an AAV helper plasmid (pAAV-RC) eliminated Rep78/68 translation, rAAV and IGF-I production. Replacement of the heterologous sequence upstream of Rep78/68 in pAAV-RC with the AAV2 endogenous p5 promoter restored translational activity to the ACG mutant, and restored rAAV and IGF-I production. Insertion of the AAV2 p19 promoter sequence into pAAV-RC in front of the heterologous sequence also enabled ACG to function as a start codon for Rep78/68 translation. The data further indicate that the function of the AAV helper construct (pAAV-RC), that is in current widespread use for rAAV production, may be improved by replacement of its AAV2 unrelated heterologous sequence with the native AAV2 p5 promoter.

**Conclusion:**

Taken together, the data demonstrate an interplay between the start codon and upstream regulatory sequences in the regulation of Rep78/68 and indicate that selective mutations in Rep78/68 regulatory elements may serve to augment the therapeutic value of rAAV vectors.

## Background

Genetic modification of cells is a promising approach to generating gene products that have therapeutic potential [1-3]. The human adeno-associated virus (AAV) has attracted attention as a vector for gene therapy because it possesses several favorable characteristics. AAV is capable of infecting dividing and non-dividing cells *in vitro *and *in vivo *and of infecting cells originating from multiple species and tissue types. No human disease has been found to be associated with AAV infection and the virus has a low immunogenicity in humans [4]. A further potential advantage for gene therapy applications is that, in the absence of a helper virus, wild type (wt) AAV can integrate into the cellular genome, an event that occurs at high frequency into a defined region on the long arm of human chromosome 19 [5-7]. This site specificity suggests that AAV may pose a low risk of insertional mutagenesis while providing the potential for long-term gene expression.

AAV DNA replication is controlled in part by four overlapping Rep proteins (Rep78, Rep68, Rep52 and Rep40) that are expressed from a single *rep *gene. Rep78 and Rep68, initiating at the p5 promoter, are expressed from unspliced and spliced transcripts, respectively. Rep52 and Rep40 are similarly produced from transcripts initiating at the downstream promoter, p19. Rep52 and Rep40 have been implicated in AAV single-stranded DNA formation and gene regulation while the two larger Rep proteins (Rep78 and Rep68) appear to convey the enzyme functions essential for AAV replication as well as regulation of viral gene expression. The capsid of the mature AAV virion is composed of three proteins that are translated from one transcript of the *cap *gene [8-10]

Three essential components are used to produce recombinant AAV (rAAV) vectors. The first is a transgene expression cassette flanked by two AAV2 inverted terminal repeats (ITRs) and constructed in a plasmid. The second is the AAV helper function of Rep and Cap proteins. The third is the adenoviral helper function provided by the products of the adenovirus E2A, E4 and VA genes. There are two commonly used methods for rAAV production. One method involves co-transfection into adenovirus-infected human embryonic kidney 293 (293) cells with two plasmids, one containing the transgene and the other providing AAV helper function. The second method involves co-transfection of 293 cells with three plasmids: the same two plasmids as noted above and a third plasmid that substitutes for the wild type (wt) adenovirus by providing E2A, E4 and VA adenoviral genes to enable viral replication. The second method offers the advantage of avoiding wt adenovirus infection and of yielding rAAV preparations that are presumed to be free of adenovirus.

Insulin-like growth factor I (IGF-I) is a cell signaling polypeptide that regulates proliferation and differentiation across a wide spectrum of cell types. Acting by both endocrine and paracrine/autocrine mechanisms, it plays a central role in the development and maintenance of multiple organs and tissues [11]. In this capacity, IGF-I has potential value in gene therapy. For this reason, it was selected for the present studies.

The potential of rAAV vectors for human gene therapy has proved elusive in part because of difficulties in producing rAAV stock with a high enough titer to be practical for therapeutic applications [12]. It is possible that Rep78/68 plays a role in determining these yields. An early report [13] suggested that an ATG-to-ACG mutation of the Rep78/68 native start codon decreased the Rep78/68 translation and increased rAAV vector production up to eightfold. The regulatory sequences upstream of Rep78/68 may also influence rAAV yield. Although these regulatory sequences may be presumed to influence Rep78/68 expression, vector production and target cell transgene expression, their role in these functions has not been elucidated.

**Figure 1:**
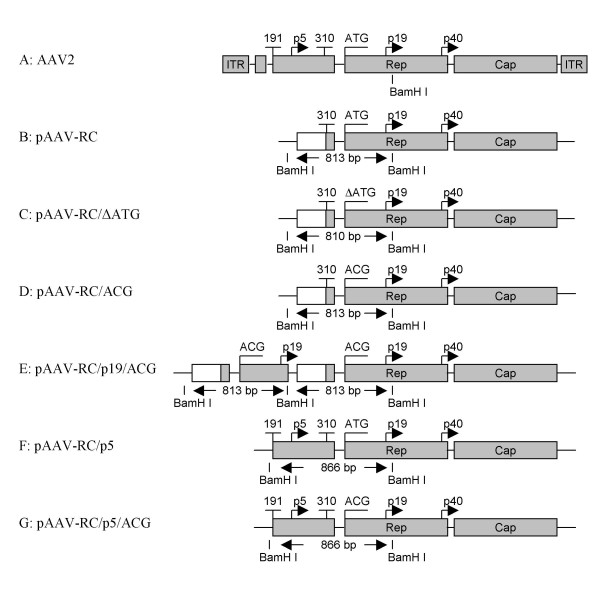
**Schematic illustration of AAV helper plasmids**. Plasmids pAAV-RC, pAAV-RC/ΔATG and pAAV-RC/ACG contain an AAV2 unrelated heterologous sequence (open boxes) upstream of Rep78/68. pAAV-RC/ΔATG has an ATG deletion at the translation start codon of Rep78/68, while pAAV-RC/ACG has an ATG-to-ACG mutation at the start codon. Plasmid pAAV-RC/p19/ACG contains an extra copy of the 813 bp BamH I fragment of pAAV-RC/ACG. Plasmids pAAV-RC/p5 and pAAV/p5/ACG contain the AAV2 endogenous p5-promoter upstream of Rep78/68 as in wt AAV2. pAAV-RC/p5/ACG has an ATG-to-ACG mutation at the translation start codon of Rep78/68. Shaded boxes represent AAV2 p5-promoter, *rep *and *cap *genes. An eleven nucleotide sequence of the p5-promoter is represented by a small shaded box before ATG or ACG in pAAV-RC, pAAV/ΔATG, pAAV/ACG and pAAV-RC/p19/ACG.

The motivation for this study was to improve target cell IGF-I production by increasing rAAV titers during rAAV preparation. The AAV Helper-Free System (Stratagene) was the only commercially available system for rAAV vector preparation. The plasmid pAAV-RC in the system contains the AAV2 *rep *and *cap *genes, coding replication proteins and viral capsid structural proteins required for AAV vector production. In wt AAV2, the Rep78/68 is regulated in *cis *by the endogenous p5 promoter. The transcription initiation site for Rep78/68 is at nt287 and the translation start codon for Rep78/68 is the ATG from nt321 to nt323 (ATG^321–323^) [9]. Sequence alignment of pAAV-RC with the AAV2 genome demonstrates that pAAV-RC contains the sequence of AAV2 genome from nt310 to nt4530, and the p5 promoter region, including the transcription initiation site for Rep78/68 in the AAV2 genome, is replaced by a heterologous promoter (Figure 1A and Figure 1B). Although the triplet ATG^321–323 ^is still present in the sequence of *rep *gene started from nt310 in pAAV-RC (Figure 1B), the promoter replacement may change the transcription intiation site of Rep78/68 and the 5' untranslated sequence of Rep78/68 transcripts, and may change the translation start codon for Rep78/68.

We tested the hypothesis that mutations in the start codon and/or upstream sequences of Rep78/68 could augment rAAV yield from 293 cells and that the resulting rAAV preparations would augment IGF-I synthesis from transduced human fibrosarcoma HT1080 cells. We tested this hypothesis by constructing a series of AAV helper plasmids containing an ATG or ACG for the Rep78/68 start codon in combination with the endogenous AAV2 p5 promoter or an AAV2 unrelated heterologous regulatory sequence. The AAV helper plasmids were compared by co-transfecting 293 cells with these plasmids and two other plasmids: pAAV-IGF-I, a plasmid carrying a therapeutic gene and pHelper, a plasmid providing an adenoviral helper function. The resulting AAV preparations were used to transduce HT1080 cells and transgene expression was assessed by measuring IGF-I production.

We found that selected modifications in the start codon and the upstream regulatory sequences of Rep78/68 significantly augmented the production of IGF-I by increasing rAAV yield.

## Results

### Effect of the Rep78/68 translation start codon in AAV helper plasmid on IGF-I Production

**Figure 2:**
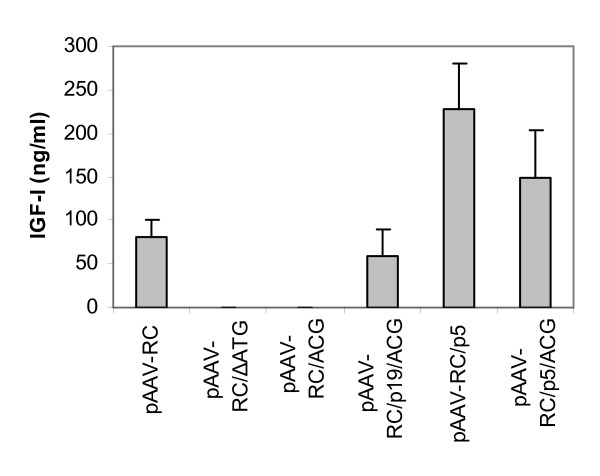
**IGF-I production from HT1080 cells transduced with rAAV-IGF-I preparations made with the designated AAV helper plasmids**. Data represent the means and SDs from four independent experiments.

There are several ATG triplets near ATG^321–323 ^in pAAV-RC that are in the same reading frame as ATG^321–323^, including ATG^447–449^, ATG^591–593 ^and ATG^627–629^. To determine whether the ATG^321–323^is a start codon for Rep78/68 translation in pAAV-RC and whether any other ATG triplet in the reading frame after ATG^321–323 ^can serve as a start codon for Rep78/68 in the absence of ATG^321–323^, we deleted ATG^312–323 ^to create pAAV-RC/ΔATG (Figure 1C). As expected, when rAAV-IGF-I was prepared with the construct pAAV-RC and used to transduce HT1080 cells, the cells secreted the transgene product, IGF-I, into the culture medium (Figure 2). In contrast, when the rAAV-IGF-I was prepared with pAAV-RC/ΔATG and used for transduction, the HT1080 cells did not produce detectable IGF-I (Figure 2). These results suggest that the ATG^321–323 ^in pAAV-RC is critical for the expression of Rep78/68, and that the other in-frame ATG triplets do not substitute for ATG^321–323^in providing this function.

To determine the role of the Rep78/68 start codon in the present system, we mutated the ATG^321–323 ^to ACG in pAAV-RC to create plasmid pAAV-RC/ACG (Figure 1D). When this plasmid was used with pAAV-IGF-I and pHelper to prepare rAAV-IGF-I, the vector preparation generated no detectable IGF-I when used to transduce HT1080 cells (Figure 2). These results were comparable to those obtained by deleting the ATG start codon.

### Effect of Rep78/68 upstream regulatory sequences in AAV helper plasmid on IGF-I production

In pAAV-RC, there is an AAV2 unrelated heterologous sequence in front of the start codon ATG^321–323 ^(Figure 1B). We postulated that the native p5 promoter may be able to regulate Rep78/68 even in the presence of the inactivating ATG-to-ACG mutation. This was tested using pAAV-RC/p5/ACG (Figure 1G) in which the heterologous upstream sequence of pAAV-RC/ACG was changed to the endogenous p5 promoter. rAAV-IGF-I was prepared with pAAV-RC/p5/ACG and used for transduction. The transduced HT1080 cells produced IGF-I (149.74 ng/ml ± 53.24, N = 4) (Figure 2). This finding indicates that the p5 promoter upstream enables the ACG triplet to function as a start codon for Rep78/68.

We further postulated that, in the presence of an ATG start codon, the endogenous p5 promoter sequence augments Rep78/68 expression in comparison to the heterologous sequence with an ATG start codon (pAAV-RC) and to the endogenous p5 promoter with an ACG codon (pAAV-RC/p5/ACG). This was tested using pAAV-RC/p5 (Figure 1F). When rAAV-IGF-I was prepared with pAAVRC/p5 and used for transduction, the transduced HT1080 cells produced IGF-I (228.04 ng/ml ± 52.37, N = 4) (Figure 2). This level of IGF-I production was significantly higher than that generated with pAAV-RC or pAAV-RC/p5/ACG (*P *< 0.001 and = 0.0044, respectively).

In wt AAV2, the two small Rep proteins (Rep52 and Rep40) are regulated by a p19 promoter sequence [9]. We hypothesized that the p19 promoter sequence could also enable Rep78/68 translation when substituted for the p5 promoter. This hypothesis was tested by inserting an extra copy of the 813 bp BamH1 fragment (ACG) into pAAV-RC/ACG to create pAAV-RC/p19/ACG (Figure 1E), containing a p19 promoter sequence upstream of the ATG-to-ACG mutation. Like pAAV-RC/p5/ACG, this construct led to IGF-I production by transduced HT1080 cells (58 ng/ml ± 30.56, N = 4) (Figure 2). The data suggest that the p19 promoter in the first copy of the 813 bp BamH1 fragment (ACG) enables the ACG triplet in the second copy of the 813 bp BamH1 fragment (ACG) to function as the start codon of Rep78/68. Interestingly, a similar construct of pAAV-RC/p19/ACG, but with the opposite orientation of the first copy of p19 promoter sequence, was ineffective and resulted in no IGF-I production (data not shown).

### Effect of AAV helper mutations on rAAV titer

The observed differences in IGF-I production by the different AAV helper constructs presumably reflects differences in the assembly of rAAV that transduces the HT1080 target cells. This presumption was tested by using real time PCR to quantify the rAAV titer generated by the different AAV helper constructs.

**Figure 3:**
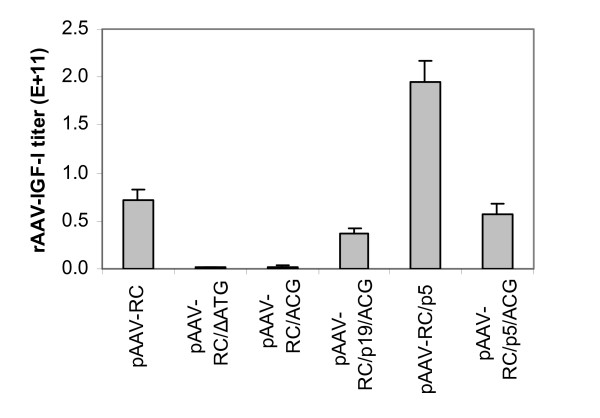
**rAAV-IGF-I titers, determined by real-time PCR, of rAAV-IGF-I preparations made with the designated AAV helper plasmids**. rAAV-IGF-I titers are expressed as viral packaged genomes/10-cm plate. Data represent the means and SDs from four independent experiments.

We found that AAV titers correlated with each preparation's transduction capacity as reflected in the production of IGF-I by transduced HT1080 cells. The highest rAAV titer was obtained when pAAV-RC/p5 was used as the AAV helper construct (Figure 3). This construct achieved 1.95 × 10^11 ^packaged genomes/10-cm plate, 2.7 fold higher (*p *= 0.001, N = 4) than with the starting helper construct, pAAV-RC. The lowest titer, 1.41 × 10^9 ^packaged genomes/10-cm plate, was obtained with pAAV-RC/ΔATG. This titer was 51.1 fold lower than that obtained with pAAV-RC (Figure 3). The other helper constructs generated rAAV titers intermediate to these values (Figure 3).

These data substantiate the use of IGF-I production as an index of rAAV titer in the preparations used to transduce the HT1080 target cells. The failure of vector preparations derived from pAAV-RC/ΔATG and pAAV-RC/ACG to elicit IGF-I production suggests that titers below 2.25 × 10^9 ^packaged genomes/10 cm plate, as observed with pAAV-RC/ΔATG (1.41 × 10^9 ^packaged genomes/10 cm plate) and pAAV-RC/ACG (2.25 × 10^9 ^packaged genomes/10 cm plate), is too low to produce detectable IGF-I transgene product when used to transduce HT1080 cells.

### Effect of AAV helper plasmids on Rep78/68 expression during rAAV preparation

**Figure 4:**
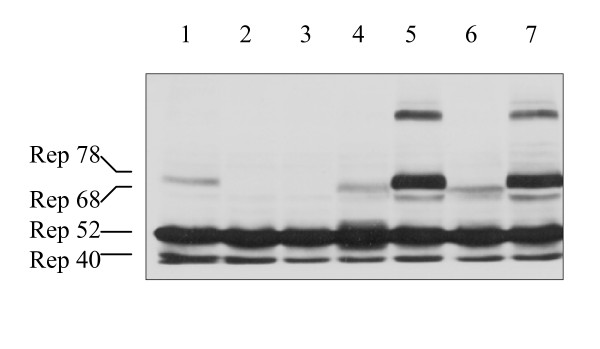
**Western blot analysis of Rep protein expression by 293 cells co-transfected with pAAV-IGF-I, the adenovirus helper plasmid pHelper and the designated AAV helper plasmids**. Plasmid pAAV-RC (lane 1), pAAV-RC/ΔATG (lane 2), pAAC-RC/ACG (lane 3), pAAV-RC/p19/ACG (lane 4), pAAV-RC/p5 (lane 5) and pAAV-RC/p5/ACG (lane 6). As a control, cells were co-transfected with the adenovirus helper plasmid pHelper and pAV1 (lane 7). Two days after the co-transfection cells were harvested, lysates were separated by SDS-PAGE and Western blotting was performed as described in Materials and Methods.

The effect of these mutations on rAAV yield, and ultimately on IGF-I transgene expression, is presumably mediated by differential regulation of Rep78/68. To test this hypothesis, we measured the effect of start codon and upstream regulatory sequences on the expression of Rep78/68 protein using western blotting analysis. We found that protein levels of Rep78 and Rep68 (Figure 4) were dependent on the construct employed and correlated with rAAV-IGF-I titer (Figure 3) and, in turn, IGF-I production by transduced HT1080 cells (Figure 2). Bands corresponding to Rep78 and Rep68 were most intense for pAAV-RC/p5 and pAV1. In both instances, Rep78 was several fold more abundant than Rep68. The Rep78 band intensity was much lower and Rep68 was barely detectable for pAAV-RC. The constructs pAAV-RC/p19/ACG and pAAV/p5/ACG generated a single predominant band between the positions of Rep78 and Rep68. This shift in relative molecular mass remains unexplained. No Rep78 or Rep68 expression was observed for either pAAV-RC/ΔATG or pAAV-RC/ACG, consistent with the failure of these constructs to generate rAAV-IGF-I preparations that yielded IGF-I. The anti-Rep antibody also identified a high molecular mass band at Mr ~140 kd that may reflect Rep78/68 dimerization [14]. The expression levels of the two small Rep proteins, Rep52 and Rep40 were similar among all six AAV helper constructs and pAV1 (Figure 4). These results demonstrate that the deletion of ATG^321–323 ^or the mutation of ATG to ACG in pAAC-RC, abolished Rep78/68 protein expression. The data further demonstrate that the p5 promoter or p19 promoter regulatory sequences upstream were able to partially restore Rep78/68 production in the presence of this inactivating mutation from ATG to ACG. These findings suggest that the effects of these mutations on vector yield and resulting transgene expression are mediated by their effects on Rep78/68 expression.

### Virus yield comparison between rAAV and wt AAV2

**Figure 5:**
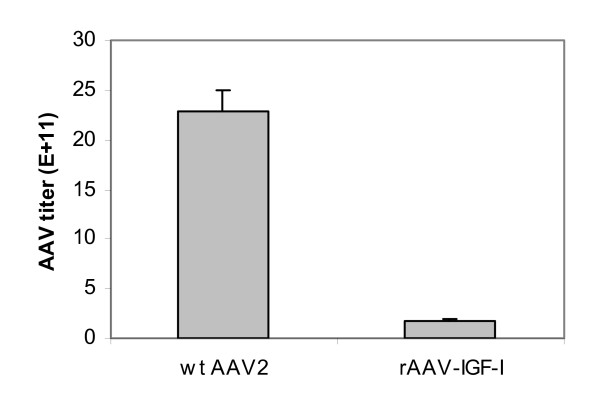
**Comparison of rAAV-IGF-I and wt AAV2 viral yield**. Wild type AAV2 was prepared by combining pAV1 (10 μg) and pHelper (10 μg) to transfect 293 cells. rAAV-IGF-I was prepared by combining the three plasmids: pAAV-RC/p5 (10 μg), pAAV-IGF-I (10 μg) and pHelper (10 μg) for transfection. rAAV-IGF-I titer and wt AAV2 titer are expressed as viral packaged genomes/10-cm plate. Data represent the means and SDs from three independent experiments.

To compare the virus yield between rAAV and wt AAV2 when AAV virus are prepared using an adenovirus free AAV preparation system, two different combinations of plasmids were used to transfect 293 cells. pAV1 with pHelper generated a wt AAV yield of 2.29 × 10^12 ^± 2.01 × 10^11 ^per packaged genomes/10-cm plate. pAAV-RC/p5 with pHelper and pAAV-IGF-I yielded an rAAV-IGF-I titer of 1.78 × 10^11 ^± 2.18 × 10^10 ^per packaged genomes/10-cm plate, a value that is significantly lower (12.9 fold difference, *P *< 0.001, N = 3) than that of wt AAV (Figure 5).

## Discussion

Difficulty in generating high titers of rAAV is an ongoing limitation in the application of rAAV technology to gene therapy. In the present studies, we investigated the mechanisms that regulate the production of rAAV. We found that the middle base in the start codon for Rep78/68 in the AAV helper construct plays a key role in this process. Specifically, the mutation of ATG^321–323 ^of Rep78/68 to ACG in plasmid pAAV-RC reduced rAAV production to a degree comparable to that obtained by deleting this codon. The associated loss of Rep78/68 protein expression in western blotting studies suggests that this effect is mediated by a reduction in Rep78/68 translation from this start codon.

These studies also clarify the role of sequences upstream of the Rep78/68 start codon in regulating rAAV production. Insertion of an AAV2 promoter sequence (p5 promoter) upstream of the ATG start codon in pAAV-RC increased production of rAAV by 2.7 fold (*p *= 0.001). This promoter also converted the otherwise non-functional ACG-containing construct to one capable of translational activity.

The regulation of Rep78/68 by upstream sequences can be subject to modulation by promoters other than the p5 promoter. When the p19 promoter was inserted upstream of the ACG start codon, translational activity was restored nearly as effectively as by the p5 promoter. The observed increase in vector yield suggests that a specific sequence such as the p5 promoter or p19 promoter upstream of the ACG is required for the recognition of the codon as a start codon for translation.

Data from the pAAV-RC/p19/ACG construct also demonstrate that the orientation of the specific sequences inserted upstream of the ACG start codon is critical. The ACG acted as start codon only when the first copy of the p19 promoter sequence was inserted in the same orientation as the second copy of p19 promoter sequence (Figure 1E). This suggests that both a specific upstream sequence such as the p5 promoter or p19 promoter and the specific sequence orientation are required for ACG to function as an initiator codon.

Six different AAV helper constructs were tested in this study. The construct pAAV-RC/p5, containing a p5 promoter upstream and an ATG start codon, achieved the highest rAAV titer. This titer was 3.4 fold higher (*p *= 0.002) than that obtained with pAAV-RC/p5/ACG, indicating that, although the p5 promoter rendered the ACG codon functional, the ATG codon performed significantly better in this context. The titer generated by the pAAV-RC/p5 construct was 2.7 fold higher (*p *= 0.001) than that obtained with pAAV-RC, suggesting that the native promoter may function more effectively in this application than the heterologous sequence used in the pAAV-RC construct. Indeed, the AAV helper construct (pAAV-RC) that is in current widespread use for rAAV production was improved by the replacement of its AAV2 unrelated heterologous sequence with the native p5 promoter. The minimal difference (5.66 × 10^10 ^versus 3.70 × 10^10 ^packaged genomes/10-cm plate, *P *= 0.344) between the titers achieved by pAAV-RC/p5/ACG and pAAV-RC/p19/ACG suggests that the p5 promoter and p19 promoter sequences are similar in their ability to function as promoters for Rep78/68 when the start codon of Rep78/68 is changed from ATG to ACG.

In this study, we compared the virus yield of rAAV and wt AAV2 using the Stratagene Helper-Free System. The titer of wt AAV2 was 12.9 fold higher (*p *< 0.001) than that of rAAV-IGF-I (Figure 5), yet the expression levels of Rep78/68 in pAV1, and pAAV-RC/p5, were very similar (Figure 4). This large difference in AAV titer in the presence of similar Rep78/68 expression suggests that factors other than Rep78/68 expression level are involved in determining AAV2 virus formation.

In the present studies, a mutation from ATG to ACG in the start codon decreased Rep78/68 protein. This finding is consistent with that of Li et al, who compared the two plasmids pAAV/Ad and pACG-2, containing ATG and ACG respectively, as Rep78/68 start codons, and noted that the change of ATG to ACG decreased translation of the two larger Rep proteins (Rep78 and Rep68) [13]. Of interest in the present study is the finding that the ATG to ACG mutation is associated with a decrease in Rep78/68 and a decrease in rAAV titer. This differs in part from an ATG to ACG associated decrease in Rep that was associated with an increase in rAAV titer [13]. This difference is unexplained, but may reflect sensitivity of results to differences in experimental design or vectors.

The two plasmids, pAAV-RC/p5 and pAAV-RC/p5/ACG, are identical with the exception that the Rep78/68 start codon in pAAV-RC/p5 is ATG while in pAAV-RC/p5/ACG it is ACG. The rAAV yield with pAAV-RC/5 was 3.4 fold higher (*p *= 0.002) than that with pAAV-RC/p5/ACG. These data indicate that, in the presence of the p5 promoter, the native ATG start codon is more effective than the mutant ACG start codon.

Initiation of translation by non-AUG codons such as ACG may be associated with a relatively low expression of the protein [15]. This was observed in the present studies. As shown in Figure 4, the levels of Rep78 protein from either pAVV-RC/p5/ACG or pAAV-RC/p19/ACG are lower than those from pAV1 or pAAV-RC/p5. During translation, nucleotides immediately flanking non-AUG codons may modulate the recognition of non-AUG start codons [15-17]. This process is unlikely to account for the observed differences in the Rep78 protein level between pAAV-RC/ACG and pAAV-RC/p5/ACG in the present study because the eleven nucleotides immediately upstream of the ACG in pAAV-RC/ACG and in pAAV-RC/p5/ACG are identical (Figure 1D and 1G). The present data suggest a similar role for sequences located more than the eleven nucleotides from the ACG codon in pAAV-RC/p5/ACG. The sequence in the p19 promoter region that similarly enabled ACG function in pAAV-RC/p19/ACG is located at least 83 nucleotides before the ACG (Figure 1D and 1E).

The titer of rAAV is relevant for gene therapy only if the rAAV is capable of serving as a vector for a therapeutic agent. In the present study, we tested the function of these rAAV vectors by incorporating the human IGF-I gene into their expression cassette. We found transduction by the rAAV preparations reliably led to secretion of the transgene protein product in proportion to the rAAV yield. These data indicate that modifications can be made in the regulatory regions of AAV helper constructs that improve rAAV production and do not disrupt transgene expression or protein product synthesis and secretion.

Taken together, these studies indicate that the regulation of rAAV production by Rep78/68 is complex. The rAAV titer is determined not only by the specific upstream regulatory sequences and translation start codons in Rep78/68, but also by specific interactions among these elements. The IGF-I production data demonstrate that specific mutations in Rep78/68 regulatory elements may serve to improve the utility of rAAV vectors for the delivery of therapeutic transgenes to target cells. Further studies will be required to optimize the value of rAAV through this and other mechanisms of rAAV synthesis and action.

## Conclusion

Our results demonstrate an interplay between start codon and upstream regulatory sequences in the regulation of Rep78/68 translation. Specifically, the p5 or p19 promoter sequence is required for the use of ACG as a start codon for Rep78/68 translation, suggesting that specific sequences are required for assisting the initiator tRNA in recognizing ACG as a start codon. We also found that the AAV helper construct that is in current widespread use for rAAV production was improved by the replacement of its AAV2 unrelated heterologous sequence with the native p5 promoter. Indeed, the native start codon, ATG, combined with the native p5 promoter, performed best of the six AAV helper constructs tested in this study. Our data suggest that decreases in the translation of the two larger Rep proteins (Rep78 and Rep68) decreases rAAV titer. However, our data also demonstrate a large difference in AAV titer between rAAV and wt AAV2 (Figure 5) in the presence of similar Rep 78/68 expression, suggesting that factors other than Rep 78/68 expression level are also involved in determining AAV2 virus formation. The data further demonstrate that rAAV vectors derived from these modified plasmids are effective in generating IGF-I overexpression in transduced target cells. These findings suggest that selective mutations in Rep78/68 regulatory elements may augment rAAV applications in gene therapy.

## Methods

### pAAV vector construction

Recombinant AAV vectors were prepared using plasmids pAAV-MCS, pAAV-IRES-hrGFP, pAAV-RC and pHelper (AAV Helper-Free System, Sratagene, La Jolla, CA). In this system, AAV2 *rep *and *cap *genes are provided by pAAV-RC.

pAAV-IRES was constructed by inserting an internal ribosome entry site (IRES) element into pAAV-MCS at BamH I and Sal I sites. The IRES element was generated by PCR using plasmid pAAV-IRES-hrGFP as a template and the two IRES primers (Table 1): IRESF and IRESR. To facilitate cloning, a BamH I site and a Sal I site were added to the 5' end and the 3' end of the PCR product, respectively. The IRES PCR product was cloned into pCR II-TOPO (Invitrogen) to create pCR II-IRES. After sequence confirmation, the IRES fragment was sub-cloned into pAAV-MCS to obtain pAAV-IRES.

**Table 1:** Primers and probes used for plasmid construction and real-time PCR

IRESF (BamH I)	5'-TCGGATCCAGCAATTCCTCGACGACTGCATAGG-3'
IRESR (Sal I)	5'-GAGTCGACCATGGTTGTGGCCATTATCATCGTG-3'
IGF-IF1 (EcoR I)	5'-CAGAATTCACAATGGGAAAAATCAGCAGTCTTCC-3'
IGF-IF2(Sal I)	5'-ACGTCGACACAATGGGAAAAATCAGCAGTCTTCC-3'
IGF-IR (Bgl II)	5'-CTAGATCTCTACATCCTGTAGTTCTTGTTTCCTG-3'
WF (BamH I)	5'-TCGGATCCGTCCTGTATTAGAGGTCACG-3'
WR (BamH I)	5'-CAGGATCCACTGCTTCTCCGAGGTAATCC-3'
WMF	5'-AACGCGCAGCCGCCACGCCGGGGTTTTACGAG-3'
WMR	5'-TCGTAAAACCCCGGCGTGGCGGCTGCGCGTTC-3'
RCMF	5'-ATCTGCGCAGCCGCCACGCCGGGGTTTTACGAG-3'
RCMR	5'-TCGTAAAACCCCGGCGTGGCGGCTGCGCAGATC-3'
RCMDF	5'-ATCTGCGCAGCCGCCCCGGGGTTTTACGAGATTG-3'
RCMDR	5'-TCGTAAAACCCCGGGGCGGCTGCGCAGATCAGAAG-3'
	
CMVF	5'-TGGGCGGTAGGCGTGTAC-3'
CMVR	5'-CGATCTGACGGTTCACTAAACG-3'
CMV probe	5'-FAM-TGGGAGGTCTATATAAGCAGAG-MGBNFQ-3'
AAV2F	5'-CAGATTGGCTCGAGGACACTCT-3'
AAV2R	5'-GTGGGCCAGGTTTGAGCTT-3'
AAV2 probe	5'-FAM-TGAAGGAATAAGACAGTGGTA-MGBNFQ-3'

We then inserted two copies of the cDNA encoding human IGF-I into pAAV-IRES. One was placed before the IRES at the EcoR I and BamH I sites and another was placed after the IRES at the Sal I and Bgl II sites. The first IGF-I fragment was generated by PCR using pCMVhIGF-I [18] as a template and primers (Table 1): IGF-IF1 (EcoR I) and IGF-IR (Bgl II). The second IGF-I fragment was generated by PCR using the same plasmid as the template and primers: IGF-IF2 (Sal I) and IGF-IR (Bgl II). The IGF-I PCR products were cloned into pCR II-TOPO (Invitrogen) and, after sequence confirmation, were sequentially sub-cloned into pAAV-IRES to obtain plasmid pAAV-IGF-I-IRES-IGF-I, abbreviated as pAAV-IGF-I.

### Construction of AAV helper plasmids

All AAV helper constructs shown in Figure 1B–G were generated from pAAV-RC. To facilitate enzymatic manipulation and DNA sequencing, mutagenesis was performed in vector pCR II. pCRII was created by removing the IRES from pCR II-IRES with restriction enzyme EcoR I and re-ligation with T4 ligase. An 813 bp BamH I fragment containing an AAV2 unrelated heterologous sequence and a part of the *rep *gene from nt310 to nt1050 of AAV2 genome including the Rep78/68 start codon (ATG) (pAAV-RC, Figure 1B), was cut off from pAAV-RC with restriction enzyme BamH I and cloned into the pCR II vector to obtain plasmid labeled as pCR II-813. The Rep78/68 start codon, ATG, was deleted in pCR II-813 by QuikChange Site-Directed Mutagenesis (Stratagene) using primers: RCMDF and RCMDR (Table 1). After sequence confirmation, the 810 bp fragment (ATG deletion) was cut out with restriction enzyme BamH I, replacing the original 813 bp BamH I fragment in pAAV-RC to create pAAV-RC/ΔATG (Figure 1C). Construction of pAAV-RC/ACG (Figure 1D), in which the ATG start codon is changed to ACG, was performed in the vector, pCR II-813 using QuikChange Site-Directed Mutagenesis with primers: RCMF and RCMR (Table 1). After sequence confirmation, the 813 bp BamH I fragment (ACG) was cut out from the pCR II vector, replacing the original 813 bp BamH I fragment in pAAV-RC. Construction of pAAV-RC/p19/ACG (Figure 1E) was performed by inserting two copies of the 813 bp BamH I fragment (ACG) at BamH I sites to replace the original 813 bp BamH I fragment in pAAV-RC.

To create a construct containing the ATG start codon and the p5 promoter regulatory sequence, the BamH I fragment within pAAV-RC/p5 (Figure 1F), which corresponds to the sequence from nt191 to nt1050 of AAV2 genome (Figure 1A), was generated by PCR using plasmid pAV1 (American Type Culture Collection, ATCC, Manassas, VA) [19] as a template and primers: WF and WR (Table 1). An extra BamH I site was added to the 5' end of the PCR product to facilitate cloning. The resulting 886 bp BamH I PCR product was cloned into the pCR II vector, creating the plasmid pCR II-866. After sequence confirmation, the 866 bp BamH I fragment was cut out of pCR II-866 and used to replace the original 813 bp BamH I fragment in pAAV-RC, creating the construct pAAV-RC/p5. To generate the construct pAAV-RC/p5/ACG (Figure 1G), the change of ATG to ACG was performed in pCR II-866 using QuikChange Site-Directed Mutagenesis with primers: WMF and WMR (Table 1). After sequence confirmation, the 866 bp BamH I fragment (ACG) was cut out from the pCR II vector and used to replace the original 813 bp BamH I fragment in pAAV-RC.

### Cell culture and virus preparation

Human embryonic kidney 293 (293) cells and human fibrosarcoma HT1080(HT1080) cells were obtained from the American Type Culture Collection. All cells were cultured in Dulbecco's minimum essential medium (DMEM) supplemented with 10% fetal bovine serum (FBS), 2 mM L-glutamine and antibiotics of 100 μg/ml streptomycin and 100 units/ml penicillin (growth medium) unless otherwise specified.

rAAV vector was prepared by calcium phosphate transfection according to the manufacturer's instructions. 2 × HBS (280 mM NaCl, 1.5 mM Na_2_HPO_4 _and 50 mM HEPES, pH 7.1) was prepared, and reagents were purchased from Sigma. Briefly, 293 cells were cultured in 10-cm cell culture plates in growth medium without antibiotics. After 2 days of culture, cells were co-transfected using 10 μg plasmid pAAV-IGF-I, 10 μg pHelper and 10 μg of one of the six AAV helper plasmids (Figure 1B–G). The plasmid DNAs were added in 1 ml of 0.3 M CaCl_2_. The DNA/CaCl_2 _mixture was rapidly mixed with 1 ml 2 × HBS and added drop-wise to the cells. For wt AAV2 preparation using the AAV helper-free system, 10 μg plasmid pAV1 and 10 μg pHelper in 0.67 ml of 0.3 M CaCl_2 _mixed with 0.67 ml 2 × HBS were used to co-transfect 293 cells. After incubation for 6 hr at 37°C, the co-transfection was stopped by replacing the media with 15 ml of growth medium. After an additional 3 days of culture, the medium was collected and stored at -80°C prior to IGF-I analysis to assess the co-transfection efficiency of each of the six constructs. The transfected cells were collected in DMEM with 2% FBS and subjected to two freeze/thaw cycles by alternating the sample between a dry ice-ethanol bath and a 37°C water bath. Cell debris was removed by centrifugation and the rAAV preparation was aliquoted and stored at -80°C until use.

### Titration of rAAV-IGF-I vector

The titers of recombinant AAV-IGF-I (rAAV-IGF-I) and wt AAV2 were determined by real-time PCR using Prism 7000 Sequence Detector System and TaqMan Universal Master Mix (Applied Biosystems, Foster City, CA) as previously described [20]. The primers (CMVF and CMVR) and probe (CMV probe), shown in Table 1, were designed to target the CMV promoter sequence for rAAV-IGF-I titer determination. The primers (AAV2F and AAV2R) and probe (AAV2 probe) in Table 1 were designed to target AAV2 *cap *sequence for wt AAV2 titer determination. The probes were 5'-end FAM and 3'-end MGB non-fluorescent quencher (MGBNFQ) labeled and custom synthesized (Applied Biosystem, Foster City, CA). Plasmid pAAV-IGF-I and pAV1 were used as the standard for quantifying rAAV-IGF-I and wt AAV2 viral titer in real-time PCR, respectively.

### Transduction of AAV-IGF-I to HT1080 cells

HT1080 cells were transduced with each rAAV-IGF-I preparation according to the AAV Helper-Free System (Stratagene) manufacturer's specifications. Briefly, HT1080 cells at a density of 8 × 10^4 ^per well were plated in 1 ml of growth medium in 24-well tissue culture plates just one day before transduction. After incubation overnight, 0.5 ml of the growth medium was removed from each well and 0.5 ml of AAV permissive medium (growth medium supplemented with 80 mM hydroxyurea and 2 mM sodium butyrate) was added to each well. The plates were returned to 37°C incubator. After the 6 hour treatment, the medium was removed and the cells were washed once with 1 ml of DMEM with 2% FBS and removed again. The cells were transduced by adding 125 μl of rAAV-IGF-I and 125 μl of DMEM with 2% FBS to each well. After 2 hour incubation, 750 μl of growth medium was added to each well and the cells were cultured overnight. To avoid interference from IGF-I present in the rAAV preparations, the medium was removed after transduction and the cells were washed with growth medium, then 1 ml of fresh growth medium was added to each well, and the cells were further cultured for two days.

### IGF-I analysis

IGF-I in the conditioned culture medium of the transfected 293 cells, in rAAV-IGF-I preparations and the conditioned medium of the rAAV-IGF-I transduced HT1080 cells was analyzed with the DuoSet ELISA Development Kit (R&D Systems, Minneapolis, MN).

### Western blotting analyses of AAV Rep proteins

Western blotting analysis of Rep proteins was performed to assess Rep protein expression following transfection using the different AAV helper constructs shown in Figure 1. Besides the six AAV helper constructs, the plasmid pAV1, which contains the full length AAV2 genome, was included for comparison. 293 cells were co-transfected with each of AAV helper plasmids mixed with plasmid pAAV-IGF-I and the adenoviral helper plasmid, pHelper. The cells were lysed and harvested two days after co-transfection. The samples were separated by SDS-PAGE and transferred to a nitrocellulose membrane. After blocking in tris buffered saline with 0.1% Tween-20 and 5% fat free milk at room temperature for two hours, the blot was incubated at 4°C overnight with an anti-Rep monoclonal antibody which recognizes all four Rep protein isoforms (clone 303.9, American Research Product, Belmont, MA).

### Statistical analysis

To compare rAAV-IGF-I yield produced with different AAV helper plasmids shown in Figure 1B–D, four independent co-transfection experiments were conducted for rAAV-IGF-I preparations and each co-transfection was performed in triplicate. To assess the IGF-I production from the HT1080 cells, four independent transductions were performed on HT1080 cells using each of the rAAV-IGF-I preparations. To compare the yield of rAAV-IGF-I made with pAAV-RC/p5 and pHelper plus pAAV-IGF-I to the yield of wt-AAV2 made with pAV2 and pHelper, three independent co-transfections were conducted and each co-transfection was performed in triplicate. Statistical analysis was performed using StatView software version 5.1 (SAS Institute, Cary, NC). Data are presented as mean ± standard deviation. The Fisher's PLSD method was used to assess differences in IGF-I production by transduced HT1080 cells and differences in rAAV-IGF-I AAV virus titer between AAV helper constructs. A student's t-Test was used to assess the difference in AAV virus titer between wt AAV2 and rAAV-IGF-I made with plasmid pAV1 and pAAV-RC/p5, respectively. *P *values less than 0.05 were considered to represent statistically significant differences.

## Abbreviations

IGF-I: Insulin-like growth factor I; rAAV: recombinant adeno-associated virus; ITR: inverted terminal repeat; Rep protein: replication protein; nt: nucleotide; wt: wild type; 293 cells: human embryonic kidney 293 cells; human fibrosarcoma HT1080 cells.

## Competing interests

The authors declare that they have no competing interests.

## Authors' contributions

SS designed the constructs and contributed to the conduct of the experiments, analysis of the data, and preparation of the manuscript. SM and RD contributed to the conduct of the experiments and data collection and analysis. SBT obtained funding for and supervised the project, and contributed to the data analysis and preparation of the manuscript.
